# Intradural Abscess of the Thoracic and Lumbar Spine

**DOI:** 10.7759/cureus.55650

**Published:** 2024-03-06

**Authors:** Nishath Rahman, Monis Ahsan, Michael Sposito, Adham Gabr, Jodi Galaydick

**Affiliations:** 1 Internal Medicine, Garnet Health Medical Center, Middletown, USA

**Keywords:** differential diagnosis of spinal epidural abscess, spinal abscess, epidural abscess, intradural abscess, laminectomy

## Abstract

Spinal abscesses are normally confined to the epidural space. Due to the anatomical seclusion of the intradural space, it is rare for the infection to spread to the area or for the cause to be idiopathic, iatrogenic in nature, or due to another phenomenon. We report a case of a 45-year-old male who was found to have a rare intradural spinal abscess two days post-laminectomy for severe central canal stenosis.

## Introduction

Spinal abscesses are a complex array of pathological manifestations that present in various forms: extramedullary, epidural, and intradural [1]. Spinal abscesses, although rare, are critical infections that cause significant morbidity. Epidural abscesses are more common than intradural abscesses, given that they usually have predisposing factors, including diabetes, immunosuppression, and invasive spinal procedures. Although primarily resulting from hematogenous dissemination of infectious agents, recent observations link an increasing number of intradural abscess cases to iatrogenic causes associated with invasive spinal procedures in particular (lumbar drains, epidural injections, etc.) [2]. They are primarily localized in the lumbar region, displaying a propensity to impact older males [3]. Intradural abscesses pose significant diagnostic hurdles because of their infrequent occurrence and elusive presentation.

## Case presentation

A 45-year-old male smoker, with a past medical history of a traumatic hip fracture at age ten for which surgical pinning has been done and a recent motor vehicle accident one month prior, presented with acutely worsening lower back pain, impaired ambulation for the past month, and one day of urinary incontinence.

**Figure 1:**
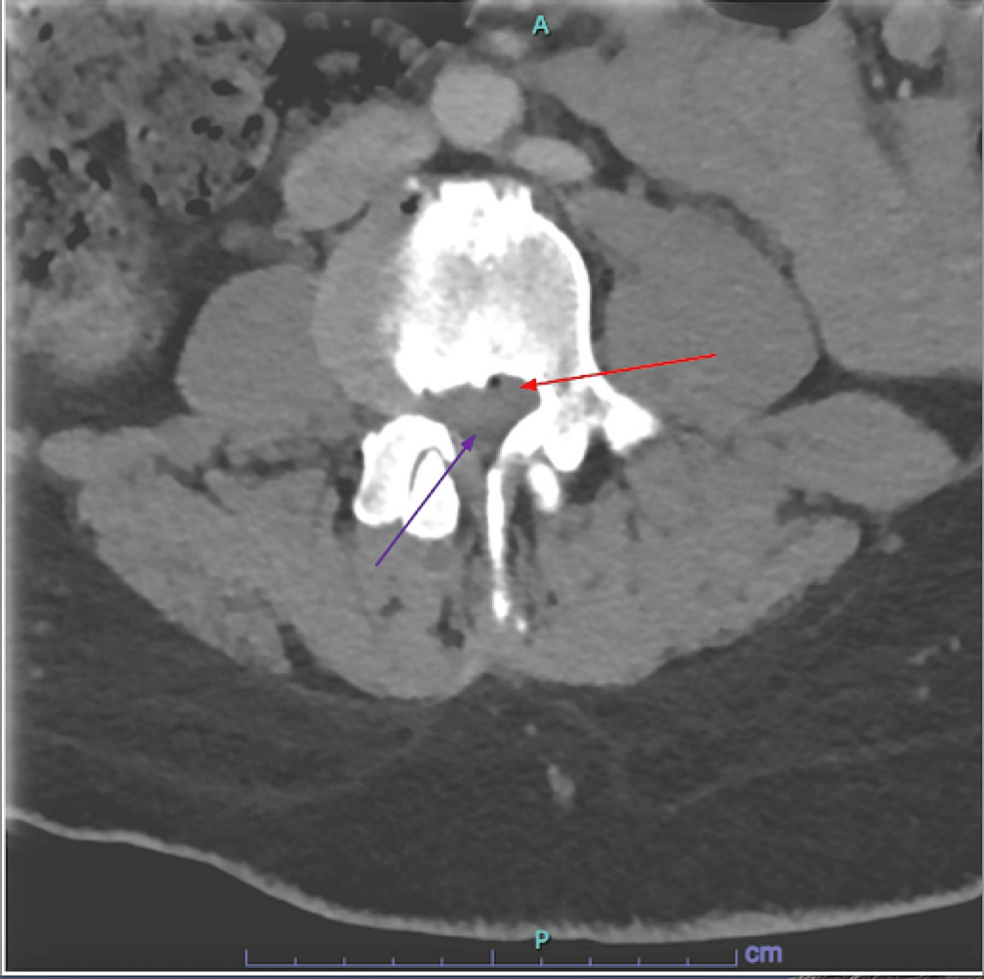
Emergency department computed tomography (CT) scan This image shows the patient's axial post-contrast CT scan in the ED, which showed a bulging disc noted by the light gray and denoted by the red arrow and a compressed spinal canal seen as dark gray and denoted by the purple arrow. The associated compressed nerves can be clinically correlated to the new-onset symptoms that are classic to cauda equina syndrome.

On presentation in the emergency department (ED), he endorsed worsening back pain with muscle spasms, a right-sided limp, and difficulty controlling his bladder. He denied headaches, loss of consciousness, seizures, blurry vision, shortness of breath, chest pain, palpitations, nausea, vomiting, or bowel incontinence. A physical exam demonstrated sensation intact throughout all extremities and intact bilateral upper extremity strength of 5/5. Left hip flexion, knee flexion/extension, and ankle dorsiflexion/plantarflexion exhibited 2/5 strength. Right hip flexion, knee flexion/extension, and ankle dorsiflexion/plantarflexion exhibited 3/5 strength. The chest X-ray was unremarkable. CT of the lumbar spine with contrast demonstrated diffuse degenerative changes most severe at L2-L3, where there was severe central canal stenosis due to an extruded disc, raising concern for cauda equina syndrome requiring urgent neurosurgical evaluation (Figure 1). Given that the patient refused an MRI on presentation, it was not done.

Neurosurgery was consulted, proceeding with L2-L5 laminectomies for severe stenosis at L2-L3. On the post-op day (POD), left hip flexion, knee flexion/extension, and ankle dorsiflexion/plantarflexion displayed 4/5 strength. Right hip flexion, knee flexion/extension, and ankle dorsiflexion/plantarflexion displayed 4/5 strength. 

On POD two, there was increased bilateral weakness in both lower extremities, with new evidence of foot drop consistent with an L2-L5 compression. The patient exhibited 3/5 hip flexion, knee flexion/extension, and 2/5 dorsiflexion/plantarflexion on the left lower extremity. The right lower extremity still displayed 4/5 hip flexion, knee flexion/extension, and dorsiflexion/plantarflexion. Findings were communicated to the neurosurgery team, and an emergent CT of the lumbar spine was completed, demonstrating persistent stenosis just inferior to the L4-L5 disc. Hematoma was not witnessed but could not be ruled out.

**Figure 2:**
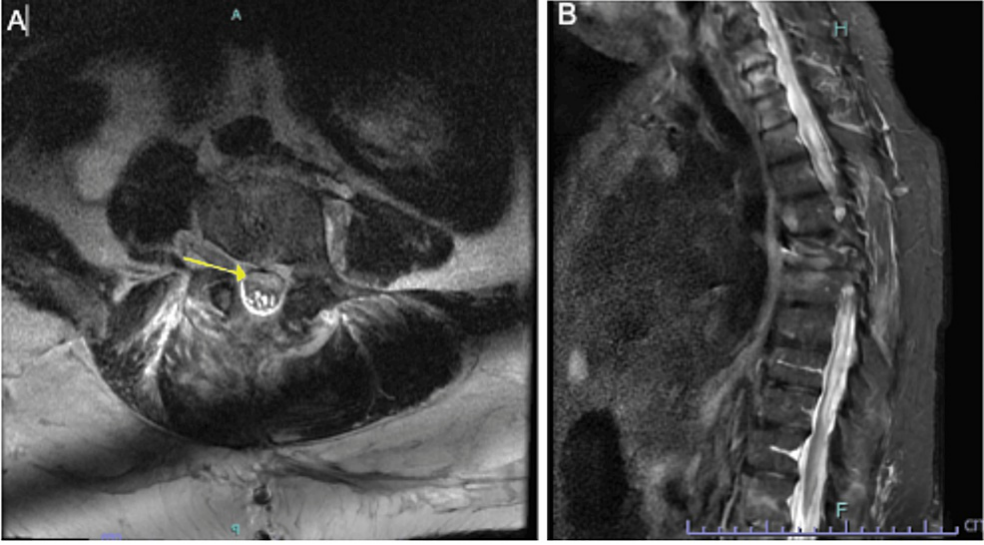
Axial magnetic resonance imaging (MRI) without contrast of the lumbar spine Figure 2A (left) denotes the clumping of the nerve roots posterior to the collection, as seen in the T2 axial image at L4. The yellow arrow denotes an area of phlegmon that is distinct from CSF and clumped roots in the spinal canal. Figure 2B (right) captures a sagittal T2 view that demonstrates the multilevel edema of the spinal cord. CSF: cerebrospinal fluid

On POD three, worsening neurological features were observed, including complete flaccidity of the left lower extremity, 0/5 strength with hip flexion, knee flexion/extension, and dorsiflexion/plantarflexion. The right lower extremity exhibited 2/5 hip flexion, 0/5 knee flexion/extension, 3/5 dorsiflexion, and 2/5 plantarflexion, consistent with nerve root involvement starting at L2. Sensation remained intact bilaterally. An MRI with and without contrast showed significant edema at several levels, arachnoiditis, and clumping of the roots in the lumbar levels in an axial view, whose etiology could not be fully delineated via imaging (Figures 2A, 2B). This was initially thought to be likely secondary to trauma from surgery.

**Figure 3:**
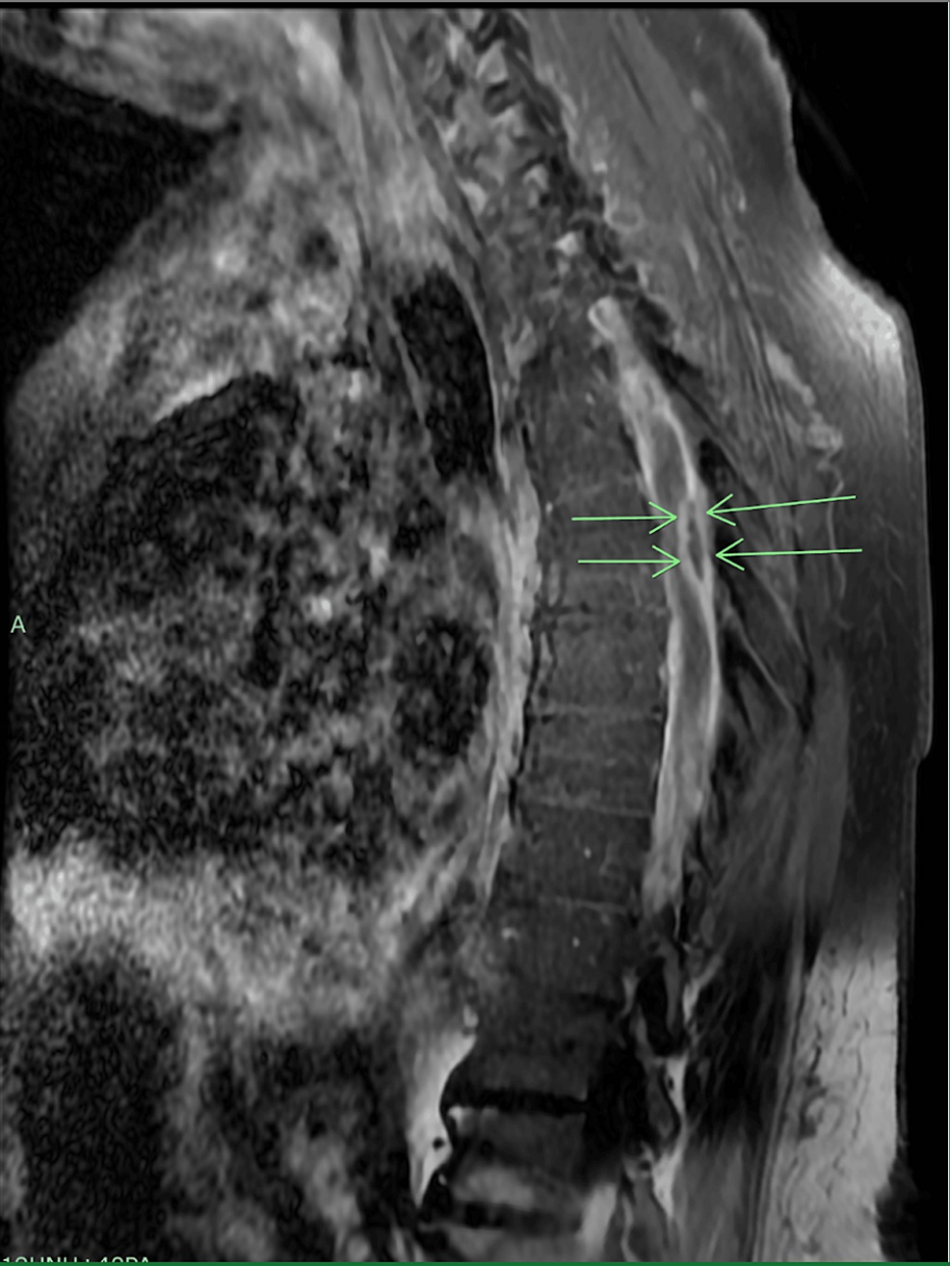
Sagittal post-contrast T1 MRI with phlegmon and spinal cord edema At the T6 level, the T1 sagittal post-contrast with fat saturation demonstrated diffuse subdural enhancement throughout the thoracic spinal canal, consistent with phlegmon. In addition, there is a 2.4x0.7 cm posterior subdural rim enhancing fluid collection at the T6 level that is consistent with intradural abscess. The abscess exerts a mass effect on the spinal cord and was clinically correlated with deficits in the patient's neurological exam.

Neurosurgery was notified and proceeded with a decompressive laminectomy. The postoperative report confirmed evidence of a suppurative process in the spinal canal between L3-L5. A thick inflammatory tissue was encountered after cutting through the dural space, beneath which an abscess cavity was found and frank purulent material was evacuated. A STAT Gram stain resulted in gram-positive cocci, and the patient was immediately started on intravenous vancomycin. The postoperative diagnosis was noted to be an intradural abscess at the level of L3-L5 that was not evident in the initial surgery. This abscess’s borders and demarcation can best be seen in pre- and post-contrast MRI images transversely in Figure 3, represented by the green arrows. Based on its location, the escalation in symptoms was clinically correlated. 

In the immediate post-op period, he was noted to have generalized upper extremity weakness that subsequently resolved after several days. Upper extremity strength was 4/5 for elbow flexion/extension and arm abduction/adduction. Bilateral lower extremity strength remained 0/5 with hip flexion, knee flexion/extension, dorsiflexion/plantarflexion, and sensation intact. C-reactive protein (CRP) was trended to demarcate antibiotic efficacy at this time and trended down from 8.1 to 0.8. A transthoracic echo also did not show any nidus of infection. Furthermore, perioperative blood cultures did not demonstrate any growth. He was sent to acute rehab with a percutaneously inserted central catheter (PICC) for continued antibiotic therapy on vancomycin with duration pending clinical course and weekly inflammatory markers.

## Discussion

This case highlights the variable and elusive clinical presentation of intradural abscesses. The case’s atypical presentation during admission lacked overt septic manifestations, but prompt and accurate neurological exams demonstrated a clinical picture of declining lower motor neuron function in the postoperative period.

Despite any signs of infection, these pronounced neurological changes served as potential red flags for pathology in the intradural space and highlighted the necessity for vigilant clinical assessment and diagnostic attentiveness in similar postoperative scenarios. Regarding the patient's mild 4/5 strength upper extremity weakness, this was transient and short-lived, lasting less than 24 hours. MRI with and without contrast of the cervical spine was considered at the time, but due to the prompt resolution of symptoms, it was not pursued. It is unclear whether the patient’s presentation was due to the motor vehicle accident one month prior or the laminectomy procedure. While it is certainly possible that the initial trauma led to the introduction of infection to the intradural space, given the acute onset of symptoms and the suspected breach of the space from the laminectomy, it is reasonable to assume that the laminectomy was the source despite the lack of confirmatory preoperative imaging, e.g., an MRI. It is also possible that this was a spontaneous introduction of the abscess into the intradural space without an underlying cause. However, even spontaneous intradural abscesses are a rare phenomenon. Of these cases, most have stemmed from the hematogenous spread of infection. 

Some rarely reported causes have been iatrogenic (i.e., epidural injection) or secondary to spondylodiscitis [4]. In this particular case, the patient was receiving steroid injections for his pain after the motor vehicle accident. It is unknown if this could have introduced the abscess to space as well. Our case is unique in the fact that abscesses were found in the thoracic and lumbar spine, most significantly in the thoracic spine, which demonstrated a mass effect. This most likely represents hematogenous spread from a distant site, as the initial procedure site was conducted in the lumbar spine (L2-L5). A previous case reported showed a similar presentation showing the hematogenous spread of intradural abscess from the lumbar to thoracic spine after uncomplicated L3-4 laminectomies with partial laminectomies of L2 and L5 for symptomatic spinal stenosis [5].

## Conclusions

Diagnosis for intradural abscesses can be challenging, often requiring a high index of suspicion, prompt imaging (MRI is the gold standard), and sometimes invasive procedures for confirmation. In this particular case, during the decompressive laminectomy, neurosurgery opened the dura in the midline to find thick inflammatory tissue and encountered the abscess cavity, from which frank purulent material was evacuated. A STAT Gram stain found gram-positive cocci. Imaging demonstrated the abscess across the thoracic and lumbar spine, but a decompressive laminectomy was done at the area of suspected significant mass effect. Timely decompressive laminectomy, abscess drainage, and antibiotics are crucial for improved outcomes. Despite advancements, spinal abscesses pose substantial risks given their acute presentation, especially when complications like postoperative infections occur, emphasizing the need for early recognition and comprehensive management to mitigate neurological sequelae.
